# Differences in gut microbial metabolism are responsible for reduced hippurate synthesis in Crohn's disease

**DOI:** 10.1186/1471-230X-10-108

**Published:** 2010-09-17

**Authors:** Horace RT Williams, I Jane Cox, David G Walker, Jeremy FL Cobbold, Simon D Taylor-Robinson, Sara E Marshall, Timothy R Orchard

**Affiliations:** 1 Gastroenterology and Hepatology Section, Division of Diabetes, Endocrinology and Metabolism, Department of Medicine, St Mary's Hospital Campus, Imperial College London, London, UK; 2 Imaging Sciences Department, MRC Clinical Sciences Centre, Hammersmith Hospital Campus, Imperial College London, London, UK; 3 Department of Immunology, University of Dundee, Dundee, UK

## Abstract

**Background:**

Certain urinary metabolites are the product of gut microbial or mammalian metabolism; others, such as hippurate, are mammalian-microbial 'co-metabolites'. It has previously been observed that Crohn's disease (CD) patients excrete significantly less hippurate than controls. There are two stages in the biosynthesis of this metabolite: 1) gut microbial metabolism of dietary aromatic compounds to benzoate, and 2) subsequent hepatorenal conjugation of benzoate with glycine, forming hippurate. Differences in such urinary co-metabolites may therefore reflect systemic consequences of altered gut microbial metabolism, though altered host metabolic pathways may also be involved.

**Methods:**

It was hypothesised that reduced hippurate excretion in CD patients was due to alterations in the gut microbiota, and not differences in dietary benzoate, nor defective host enzymatic conjugation of benzoate. 5 mg/kg sodium benzoate were administered orally to 16 CD patients and 16 healthy controls on a low-benzoate diet. Baseline and peak urinary hippurate excretion were measured.

**Results:**

Baseline hippurate levels were significantly lower in the CD patients (p = 0.0009). After benzoate ingestion, peak urinary levels of hippurate did not differ significantly between the cohorts. Consequently the relative increase in excretion was significantly greater in CD (p = 0.0007).

**Conclusions:**

Lower urinary hippurate levels in CD are not due to differences in dietary benzoate. A defect in the enzymatic conjugation of benzoate in CD has been excluded, strongly implicating altered gut microbial metabolism as the cause of decreased hippurate levels in CD.

## Background

The pathogenesis of the inflammatory bowel diseases (IBD), Crohn's disease (CD) and ulcerative colitis (UC), is thought to involve a genetically-determined, abnormal host immune response to an environmental stimulus, which is likely to be bacterial [1]. There is compelling evidence that dysbiosis of the commensal enteric microbes plays an important role in the pathogenesis of these diseases [2]. Urinary metabolite levels are strongly influenced by differences in the intestinal microbiota, since both gut bacterial metabolism, and shared metabolism by the host and bacterial species ('co-metabolism'), generate specific metabolic products [3]. Such metabolites may therefore be used as markers of microbial metabolic activity, reflecting systemic, functional differences. This application of urinary metabolic profiling avoids the technical difficulties, and methodological differences, found in molecular studies of the intestinal microbiota in IBD, which have contributed to often discrepant findings [4].

It has previously been shown that urinary levels of the host-bacterial co-metabolite, hippurate, are significantly lower in IBD patients when compared to healthy control individuals, most significantly in those with CD (p < 0.0001) [5]. These differences were independent of medication, disease location and disease activity.

Differences in urinary co-metabolites may reflect altered gut microbial metabolism, though altered host metabolic pathways may also be implicated. This study was undertaken to investigate the biosynthesis of hippurate in IBD, and specifically to clarify the influence of the gut microbiota on urinary hippurate excretion.

The origins of hippuric acid have been investigated since the compound was first identified in urine by Liebig in 1829 [6]. By the early 20th century, it had been established that in man, hippurate is the product of the conjugation of benzoate with glycine [7]. Later investigations revealed that this conjugation occurs via the formation of an intermediate, benzoyl CoA [8], and that it takes place in the mitochondria [9] of the liver and the kidney [10]. Animal experiments have shown that urinary hippurate excretion is modulated according to the composition of the intestinal microbiome [11,12].

**Figure 1:**
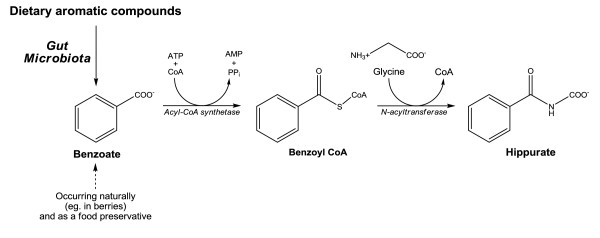
**The synthesis of hippurate in man**.

The biosynthesis of hippurate is shown diagrammatically in (Figure 1). It is excreted at millimolar concentrations in human urine.

Benzoate, a simple carboxylic acid, is produced from the microbial degradation of dietary aromatic compounds in the intestine, such as polyphenols, purines, and aromatic organic acids and amino acids [13-15]. Consequently, hippurate excretion has been shown to increase following the intake of black, green and chamomile tea [16,17] as well as a diet rich in fruit and vegetables [18]. Benzoate itself is also present in various foodstuffs and drinks. It is present naturally in most berries, fruits and fermented dairy products [19]. Due to their antimicrobial properties, both benzoic acid and benzoate salts may be used as preservatives. While common additives around the world, in the United Kingdom these compounds are little used, though may be found in soft drinks, sauces and reduced-sugar jams [20].

Early reports noted that hippurate production after benzoate ingestion appeared to eliminate nitrogen that would otherwise have been excreted as urea [7]. This has been applied clinically in the management of hepatic encephalopathy, at a dose of 10 g per day [21], and in the treatment of patients with urea cycle enzymopathies in therapeutic dosages of 150-500 mg/kg body weight per day [22]. Consequently, more recent experiments have investigated the pharmacokinetics of benzoate in relation to its use in the treatment of disease, concluding that the substrate exhibits Michaelis-Menten kinetics [23]. The saturable nature of this metabolism at very high doses, due to limitations in the availability of glycine, has been demonstrated [23,24].

The metabolism and elimination of low doses of benzoate occurs rapidly, with the peak response of urinary hippurate excretion within 1-2 hrs of ingestion [25], for at low doses saturation of the metabolic pathway does not occur. A World Health Organization review suggested a No-Observed-(Adverse)-Effect-Level of about 500 mg/kg body weight [26]. This incorporated an uncertainty factor of 100, and recommended a provisional, chronic acceptable intake of 5 mg/kg body weight per day.

### Rationale for the study

Elucidating the metabolism and biosynthesis of hippurate may provide insights into intestinal microbial dysbiosis in IBD.

There are two steps to the production of urinary hippurate from dietary sources. Firstly, the metabolism of dietary aromatic compounds to benzoate by the gut microbiota and secondly, the subsequent conjugation of benzoate with glycine to form hippurate. Hippurate excretion is reduced in IBD cohorts [5]. Since the most significant differences were observed in the CD cohort, it was these patients rather than UC patients who were chosen for inclusion in the current study. It is known that the gut microbes in CD differ from the healthy population [27], and so it was hypothesised that an alteration in the intestinal microbiota was responsible for the variation in hippurate excretion.

To confirm that the reduced urinary hippurate was not due to differences in dietary benzoate, foods known to contain high concentrations of natural benzoate, its precursors, or benzoate as a preservative, were excluded from the diet.

In order to exclude a defect in benzoate conjugation in IBD as the cause of reduced urinary hippurate levels, sodium benzoate was administered orally to groups of healthy control individuals and CD patients. It was hypothesised that, after benzoate ingestion, the increase in urinary hippurate would be similar in the two cohorts, and so the increase would be relatively *greater *in the CD population, whose baseline levels of hippurate are lower.

## Methods

### Subjects

The study was approved by the St. Mary's Research Ethics Committee (Ref 08/H0712/35) and all participants gave written, informed consent.

Patients with CD and healthy individuals were invited to participate: vegetarians and those on a therapeutic diet for IBD were considered ineligible, due to the likely influence on urinary metabolite levels [18]. Patients with significant comorbidity or a history of orofacial granulomatosis (the treatment of which may involve a benzoate-free diet) were excluded from the study [28]. Individuals with an intercurrent illness, who were pregnant, or who were taking antibiotics, pre- or probiotics were also excluded.

Studies have shown that a minority of patients with CD has increased intestinal permeability, and it has been suggested that this is correlated with disease activity [29]. Urinary hippurate levels were in fact lower in the previously studied CD cohort [5], making increased permeability unlikely as the cause for the observed differences. The differences in urinary hippurate also persisted whether disease was active or quiescent. Nonetheless, all of the CD participants in the current experiment were studied in remission as defined by the Harvey-Bradshaw Index [30].

### Dosage of sodium benzoate used for the study

To determine the dose of benzoate to be administered for the study, a review of the literature was undertaken [7,23-25,31] and a pilot experiment was carried out in a healthy control individual, using nuclear magnetic resonance (NMR) spectroscopy to quantify urinary hippurate as described previously [5].

It was established that, after a dose of the WHO recommended maximum daily intake of benzoate (5 mg/kg body weight), there was an easily detectable increase in urinary hippurate excretion at 1 hr post-dose, and that by 2 hrs post-dose the urinary hippurate concentration had decreased to near baseline values. Hence a dose of 5 mg/kg body weight was chosen.

## Study Protocol

### Dietary restrictions

After agreeing to participate, each individual was asked to avoid, for 24 hrs, specific drinks and foodstuffs known to contain high levels of benzoic acid/sodium benzoate, or significantly to influence hippurate synthesis through the metabolism of other organic acids. These were: black, green or herbal teas, fruit and carbonated drinks, berries, pickles and yoghurt.

Subjects were asked to provide a dietary and lifestyle history, as previously described [5], to ensure that there were no significant differences between the cohorts which could influence results.

### Sodium benzoate administration

Participants were asked to give a random, mid-stream urine sample between 10.00 and 14.00 hrs. A dose of 5 mg/kg body weight sodium benzoate was then administered in the form of '*Amzoate *' Sodium Benzoate Oral Liquid (sugar free, Dales Pharmaceuticals Ltd, UK for Special Products Ltd, UK) and subjects were asked to provide urine samples at 1 and 2 hrs post-dose. During this period, they were requested not to eat or drink.

### ^1^H NMR spectroscopy of urine samples

A standard technique for the preparation of urine samples was used [5]. This involved mixing 400 μ L of each sample with 200 μ L of 0.2 M phosphate buffer, pH 7.35, to stabilize pH. Any precipitates were removed by subsequent centrifugation. 500 μ L of supernatant were then mixed with 50 μ L of 3-trimethylsilyl-(2,2,3,3-^2^H_4_)-1-propionate (TSP)/D_2_O solution. The TSP served as an internal chemical shift reference (δ 0.00 ppm) and the D_2_O provided a field lock.

Samples were analysed blinded, in a random order. 1 D spectra were acquired using a JEOL 500 MHz Eclipse+ NMR spectrometer and a standard protocol with water presaturation. The spectral width was 15 ppm and the number of data points acquired was 64 K. A pulse-collect sequence was used with a 90° pulse angle. The acquisition time was 8.74 s. A relaxation delay of 10 s (total repetition time 18.74 s) ensured full relaxation between data collects. The 16 data collects were summated.

Spectra were processed using the KnowItAll Informatics System v7.8 (Bio-Rad, Philadelphia, USA). Free induction decays were zero-filled by a factor of two and multiplied by an exponential window function with a 0.3 Hz line-broadening factor prior to Fourier transformation. Spectra were phased and a baseline correction applied.

### Statistical analysis

Resonances corresponding to hippurate (δ 7.83 ppm, doublet) were integrated and normalized to the sum of the total spectral integral. Values were expressed as a relative index, to the total spectral integral, corrected for the number of contributory protons (2H); values were also expressed as mmol/mol creatinine (δ 4.07 ppm, singlet, 2 H).

Hippurate levels in the CD and control cohorts, pre-dose, were compared using the Mann-Whitney U test. Comparisons between the paired samples of each individual were made using the Wilcoxon signed rank test. Peak hippurate excretion values for the CD and control cohorts (1 hr post-dose) were compared using the Mann-Whitney U test.

The peak hippurate excretion value for each participant at 1 hr post-dose was subtracted from the baseline value, and the resulting figure, representing the absolute difference in hippurate level, was compared between the CD and control cohorts. The increase in hippurate level as a percentage change was also calculated.

Dietary and lifestyle data from the cohorts were analysed using the Mann-Whitney U test for continuous variables and Fisher's exact test or chi-square test (as appropriate) for categorical variables.

## Results

### Subject groups

Subject demographics, and details of disease for the CD cohort [32], are shown in (Table 1). Two cohorts of 16 participants were recruited. The median age of the CD cohort was non-significantly higher than the control cohort. Three of the CD patients were taking no medication.

**Table 1:** Participant characteristics

	Crohn's disease	Healthy controls
Number (Male/Female)	16 (7/9)	16 (9/7)

Median [range] age (years)	42 [22-66]	33 [22-62]

Disease location	L1: 2	-
	L2: 6	-
	L3: 8	-

Previous bowel resection	6	

L1 = ileal disease, L2 = colonic disease, L3 = ileocolonic disease [32].

There were no significant differences between the groups with regards to dietary constituents, smoking, alcohol intake or exercise. Female subjects were matched for hormonal status.

None of the participants reported side-effects of the benzoate ingestion.

### Sample collection

For all subjects, baseline (pre-dose) and peak response samples, at 1 hr, were collected. In both cohorts, excretion of urinary hippurate was highest at 1 hr post-dose, and had decreased by 2 hrs. The baseline and peak response values were used for analysis of conjugating ability.

### Baseline hippurate levels

**Figure 2:**
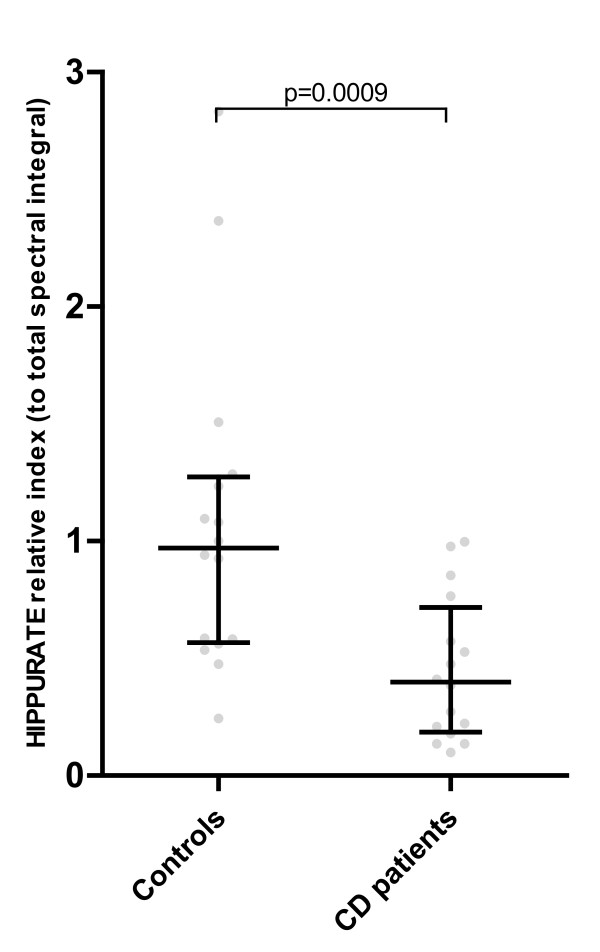
**Baseline urinary hippurate levels**. Expressed relative to total spectral integral. Median and interquartile ranges shown; p = 0.0009, Mann-Whitney U test.

A comparison was made between hippurate levels in the CD and control cohorts at baseline (ie. on the diet as described above) to negate the possible effect of foods and drinks containing either endogenous benzoate, or containing benzoate as a preservative, on hippurate excretion. The results are shown in(Figure 2). As hypothesised, significant differences were observed between the cohorts. The dietary restrictions of this study in fact had little impact on urinary hippurate values: the median values (relative to the total spectral integral) in this study for the CD and control cohorts were 0.39 u and 0.97 u respectively, compared to 0.38 u and 1.04 u in the previous study [5].

Expressed as mmol/mol creatinine, results of a similar statistical significance were obtained, hippurate levels being lower in the CD cohort: median (interquartile range) 113 (61-182) mmol/mol creatinine vs. 242 (143-365) mmol/mol creatinine, p = 0.007.

### Hippurate excretion after benzoate ingestion

**Figure 3:**
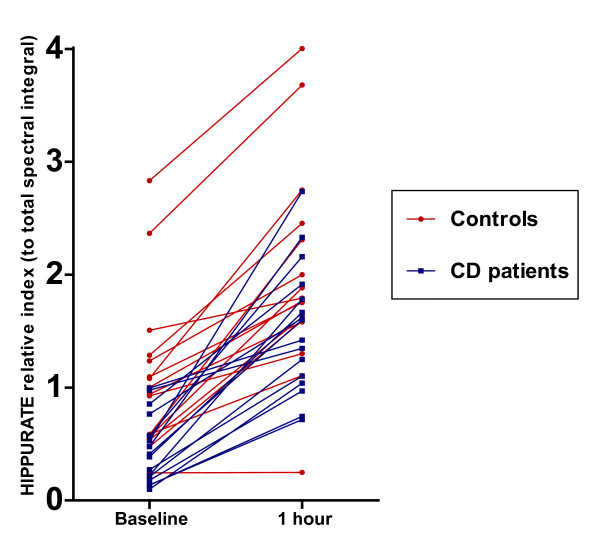
**Increase in hippurate excretion 1 hr after benzoate ingestion**.

**Figure 4:**
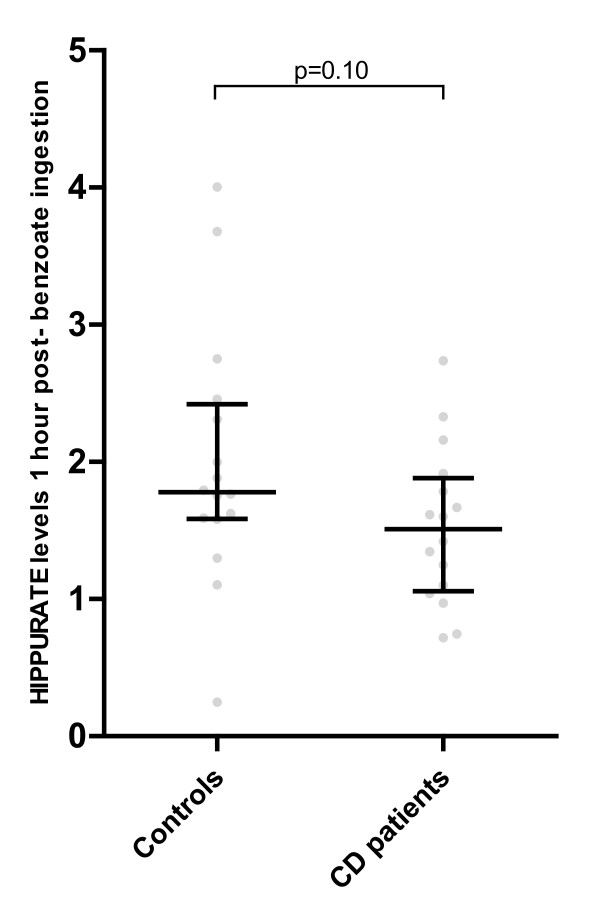
**Peak urinary hippurate levels 1 hr after benzoate ingestion**. Expressed relative to total spectral integral. Median and interquartile ranges shown; p = 0.10, Mann-Whitney U test.

**Figure 5:**
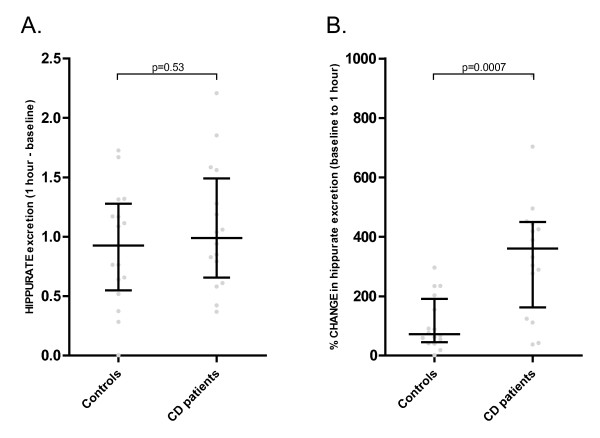
**Differences between control and CD cohorts in hippurate excretion, 1 hr after benzoate ingestion**. Median and interquartile ranges shown. A. Absolute difference, expressed as a relative index, to the total spectral integral. B. Percentage change.

The increase in hippurate excretion after the ingestion of 5 mg/kg benzoate for the CD and control groups is shown in (Figure 3). It can be seen that the baseline values for the control cohort were significantly greater than those for the CD cohort, and that the values for peak excretion at 1 hr increased more dramatically for the CD cohort. The peak excretion values did not differ significantly between the healthy control subjects and CD patients (Figure 4), nor were there significant differences in the absolute difference in hippurate excretion (calculated as the value at baseline subtracted from the value at 1 hr, (Figure 5A). As may be inferred from these figures, the percentage change in hippurate excretion was significantly greater in the CD than the control cohort (p = 0.0007, Figure 5B).

Expressed as mmol/mol creatinine, results were very similar: peak excretion values did not differ (p = 0.34), nor did the absolute differences in hippurate excretion (p = 0.25). The percentage change in hippurate excretion was greater in the CD cohort (p = 0.0006).

## Discussion

This experiment has confirmed the previous finding of reduced urinary hippurate levels in CD. It has demonstrated that the difference in urinary hippurate between CD and controls is not due differences in ingested benzoate, and that patients with CD do not have an intrinsic defect in the conjugation of benzoate with glycine. This implicates alterations in the gut microbiota in the observed differences in hippurate excretion.

The influence of diet on the excretion of urinary hippurate has been demonstrated in previous studies [17,18,33], emphasising the need for a careful evaluation of dietary components when analysing urinary metabolic data in the context of disease. As shown in (Figure 2), the reduced excretion of hippurate in CD patients persisted when foods known to contain high levels of benzoic acid/sodium benzoate, or to influence hippurate synthesis through the metabolism of other organic acids, were excluded from the diet. This finding supports the contention that it is the generation of benzoate via the intestinal microbial metabolism of dietary aromatic compounds that primarily influences the differences in urinary hippurate between the cohorts.

To ensure that the reduced levels of urinary hippurate excretion in CD were not due to an intrinsic defect in hippurate metabolism, this experiment investigated the ability of a CD cohort to conjugate benzoate with glycine, in comparison to a healthy control cohort. A dose of 5 mg/kg of sodium benzoate was administered to all participants, and it was found that the peak excretion of the metabolic product, urinary hippurate, occurred at 1 hr post-dose. There have been no published studies investigating the excretion of hippurate after the ingestion of this dose of benzoate, but inspection of the data from two previous studies, one using much higher doses [23] and another using lower doses [25] suggests that these results are compatible.

Kubota and Ishizaki, after studying the excretion of hippurate after extreme doses of 40, 80 and 160 mg/kg sodium benzoate, deduced that the biotransformation of benzoic to hippuric acid follows saturable, or Michaelis-Menten kinetics, with a maximum rate of biotransformation of 23 mg.kg^-1^.h^-1^[23]. At a dose of 5 mg/kg, as employed in the current study, the metabolically active enzymatic components of the pathway and endogenous glycine, rather than the substrate (benzoate), are in great excess. Consequently, in the presence of an intact enzymatic conjugation pathway, the CD cohort had a relatively higher production of hippurate because baseline levels were significantly lower: as seen in (Figure 5B), this was the salient finding of the study.

Thus, although the baseline hippurate levels were lower in the CD cohort, the absolute difference in hippurate excretion from baseline to peak excretion (1 hr post-benzoate administration) was not significantly different between the cohorts, and the percentage change in hippurate excretion of the CD patients was significantly greater than that of the controls. These results demonstrate that there is no deficit in the conjugation pathway in CD patients.

The concentration of urinary hippurate has been shown to be modulated according to the composition of the intestinal microbiota [11,12]. The findings of this study provide additional evidence for the systemic effects of an altered gut microbiome in IBD, notably identifying a functional metabolic consequence of the dysbiosis.

A recent study in healthy individuals has given further insights into the powerful influence exerted by the gut bacteria in the determination of human metabolic phenotypes [34]. A reduction in *Clostridia *spp. has been consistently shown in IBD, and particularly CD [4]; Li *et al. *[34] found a positive association between *Clostridia *spp. and hippurate levels, which may account for the reduced hippurate levels in our study. Future studies correlating the urinary metabolic profiles of IBD patients with molecular analysis of their gut microbiota would be of great interest, representing an avenue for further research.

Recently, Brahmachari *et al. *have investigated the anti-inflammatory properties of benzoate in the context of glial cell activation [35]. In experimental animals, benzoate, but not formate, inhibited glial activation of NFκB and expression of inducible NO synthase and pro-inflammatory cytokines. Reduced synthesis of benzoate by the intestinal microbiota in IBD may thus be implicated in the pathogenesis of the disease; further experiments in models of intestinal inflammation are warranted.

## Conclusions

In conclusion, the biosynthesis and excretion of urinary hippurate has been investigated in cohorts of CD patients and healthy control individuals. The data presented provide strong evidence for the pivotal role of the gut microbiota in the generation of urinary hippurate and, moreover, their influence on the differences in hippurate excretion between CD patients and healthy control individuals.

## Competing interests

The authors declare that they have no competing interests.

## Authors' contributions

HRTW, IJC, JFLC, SDT-R, SEM and TRO were responsible for the study design and co-ordination. Samples were collected and prepared by HRTW and DGW. NMR spectroscopy was performed by IJC, HRTW, DGW and JFLC. HRTW analysed the data with IJC and TRO. HRTW drafted the report. All authors participated in critical revision of the report.

All authors have approved the final draft submitted.

## Pre-publication history

The pre-publication history for this paper can be accessed here:

http://www.biomedcentral.com/1471-230X/10/108/prepub
